# Supplementary material for: Pathogenic KRAS variants disrupt structure and dynamics: Insights from integrated computational analyses
Source: PLoS One. 2026 Feb 11;21(2):e0341219. doi: 10.1371/journal.pone.0341219 (PMC12893532; doi:10.1371/journal.pone.0341219)
Supplement: S1 Fig — (DOCX) [file pone.0341219.s001.docx]

**
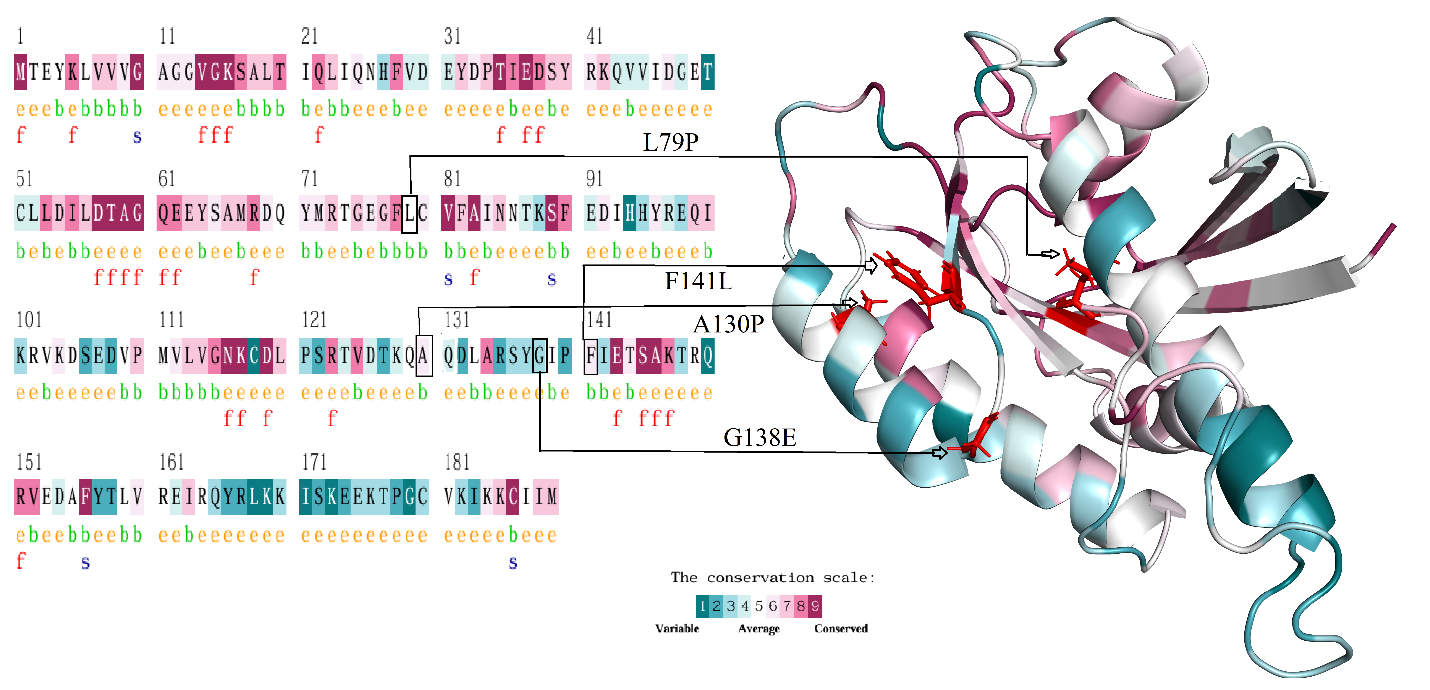
S1 Fig. Consurf evaluation of KRAS nsSNPs illustrating critical regions for maintaining the protein functionality and structural stability.**
