# Supplementary material for: Pathogenic KRAS variants disrupt structure and dynamics: Insights from integrated computational analyses
Source: PLoS One. 2026 Feb 11;21(2):e0341219. doi: 10.1371/journal.pone.0341219 (PMC12893532; doi:10.1371/journal.pone.0341219)
Supplement: S2 Fig — (A-C) Wild, (B-D) Mutant. (DOCX) [file pone.0341219.s002.docx]

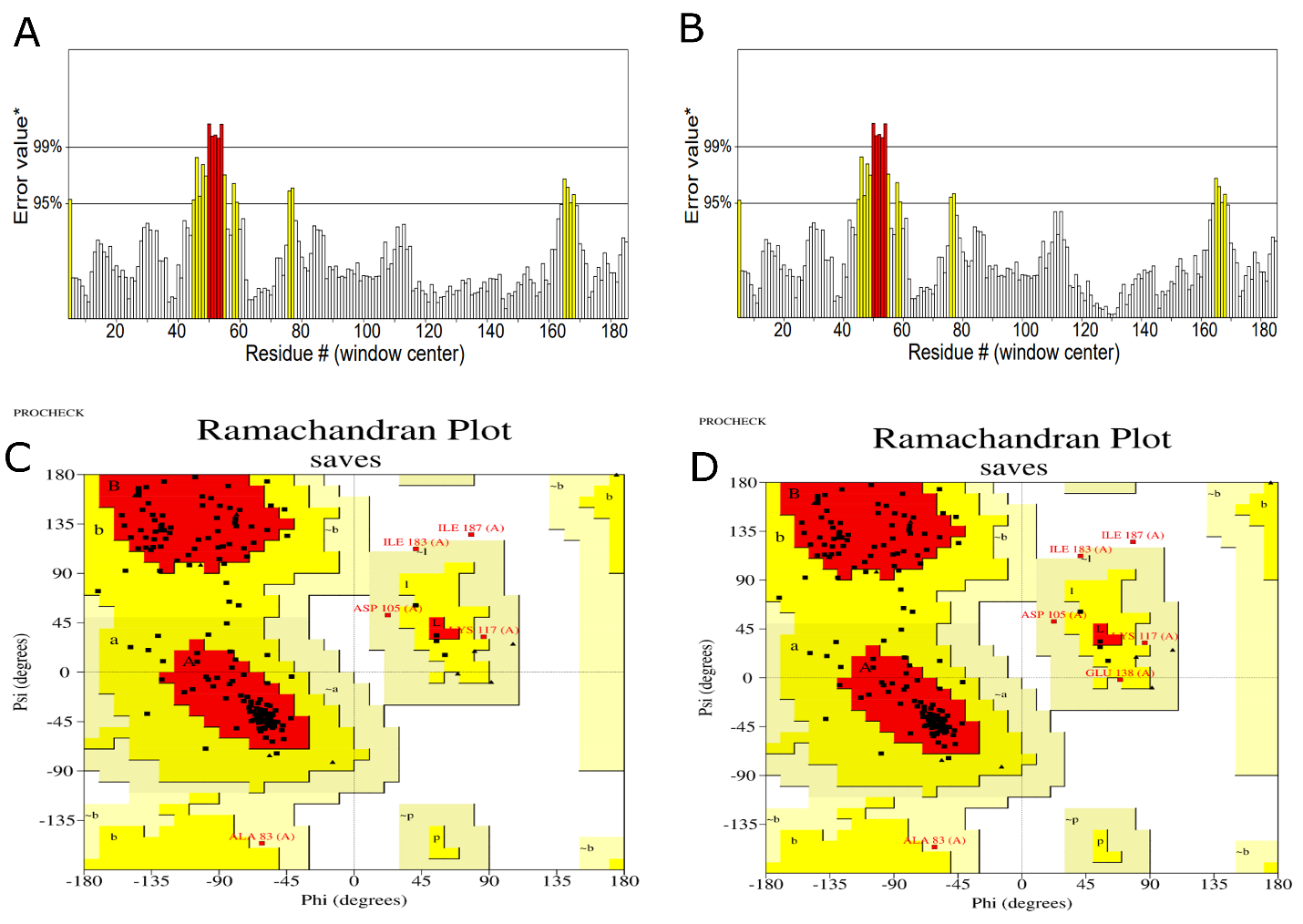


**S2 Fig. Validation of wild and mutant KRAS protein models via ERRAT and Ramachandran plots.** (A-C) Wild, (B-D) Mutant
