# Supplementary material for: Pathogenic KRAS variants disrupt structure and dynamics: Insights from integrated computational analyses
Source: PLoS One. 2026 Feb 11;21(2):e0341219. doi: 10.1371/journal.pone.0341219 (PMC12893532; doi:10.1371/journal.pone.0341219)
Supplement: S1 Table — (DOCX) [file pone.0341219.s003.docx]

**Table S1. Docking study for wild and mutant KRAS protein-ligand complexes, showing binding scores, key residues and interaction distances (Å).**

| **Ligands** | **Protein Type** | **Key Interacting Residues** | **Hydrogen Bonds (Å)** | **Hydrophobic / π–π Interactions** | **Binding Affinity (kcal/mol)** |
| --- | --- | --- | --- | --- | --- |
| **Salirasib** | Wild | Ala134, Arg135, Met189 | 2.6 (Arg135), 3.0 (Ala134) | Met189 (hydrophobic) | -6.0 |
|  | Mutant | Ala134, Arg135, Pro140, Leu141, Arg151 | 2.4 (Arg135), 2.8 (Ala134) | Leu141, Pro140, Arg151 (hydrophobic) | -6.3 |
| **Soforasib** | Wild | Arg135, Pro140, Arg151 | 2.5 (Arg135) | Pro140, Arg151 (π–alkyl) | -5.4 |
|  | Mutant | Gln131, Arg135, Leu141, Arg151, Met189 | 2.3 (Arg135), 3.1 (Gln131) | Leu141,Arg151 Met189 (hydrophobic) | -5.2 |
| **Garsorasib** | Wild | Ile142, Arg151, Asp154 | 2.8 (Asp154) | Ile142, Arg151 (hydrophobic) | -5.0 |
|  | Mutant | Gln131, Ala134, Arg135, Pro140, Leu141, Arg151 | 3.0 (Gln131), 2.8 Ala134, 2.6 (Arg135), | Pro140, Leu141, Arg151 (π–π, hydrophobic) | -5.1 |
