# Supplementary material for: Pathogenic KRAS variants disrupt structure and dynamics: Insights from integrated computational analyses
Source: PLoS One. 2026 Feb 11;21(2):e0341219. doi: 10.1371/journal.pone.0341219 (PMC12893532; doi:10.1371/journal.pone.0341219)
Supplement: S3 Fig — (DOCX) [file pone.0341219.s004.docx]

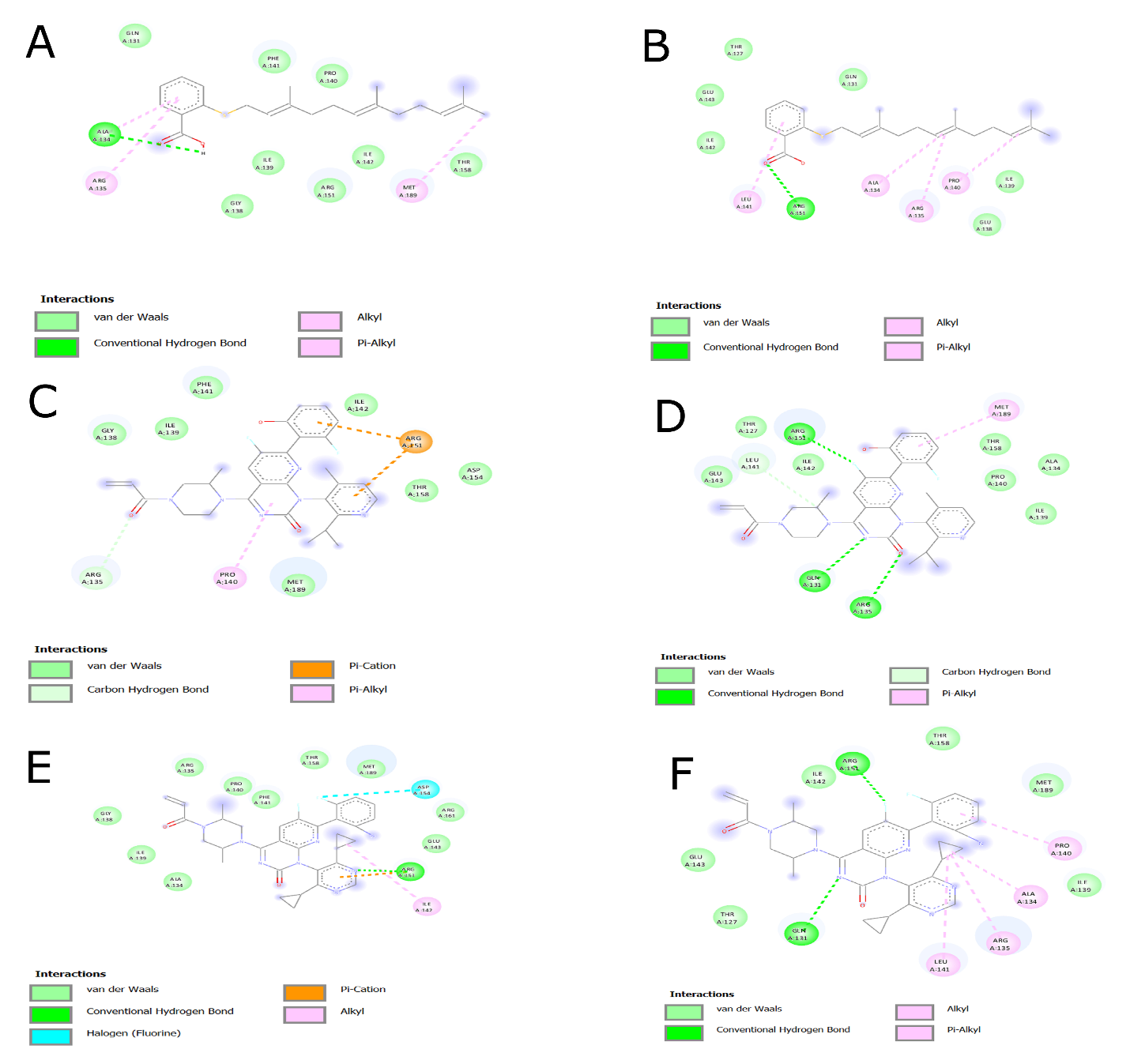


**S3 Fig. (2D) schematic representation illustrating critical interactions between the protein-ligand, highlighting biding site residues and interaction types**
