# Supplementary material for: Pathogenic KRAS variants disrupt structure and dynamics: Insights from integrated computational analyses
Source: PLoS One. 2026 Feb 11;21(2):e0341219. doi: 10.1371/journal.pone.0341219 (PMC12893532; doi:10.1371/journal.pone.0341219)
Supplement: Attachment — Submitted filename: Response to Reveiwers.docx [file pone.0341219.s006.docx]

**Pathogenic KRAS Variants Disrupt Structure and Dynamics: Insights from Integrated Computational Analyses**

Dear Editor,

Thank you very much for the reviews of this manuscript. We have described below our responses to the comments from reviewer 1, 2 and 3, followed the suggestions of reviewers and editor. The changes made in the revised manuscript are highlighted yellow.

**Reviewer 1**

**Comments 1:**

The background should better highlight the clinical significance of KRAS mutations and why nsSNP analysis is crucial compared to other oncogenic drivers.

Response:

We thank the reviewer for this valuable suggestion. In response, we have revised the introduction to better emphasize the clinical significance of KRAS mutations, noting their association with poor prognosis, tumor aggressiveness, and resistance to targeted therapies, and have highlighted why analysis of nsSNPs in KRAS is particularly important compared to other oncogenic drivers. (Page Number 2, Line number 55-60)

**Comments 2:**

Clarify the novelty of this study by distinguishing it from previous KRAS in-silico mutation studies.

Response:

We thank the reviewer for this insightful comment. In response, we have clarified the novelty of our study by highlighting that, unlike previous studies which were often limited to small datasets or single predictive algorithms, our work integrates multiple bioinformatics tools, structural modeling, and stability analyses to comprehensively evaluate deleterious nsSNPs in KRAS, providing a deeper molecular understanding of KRAS missense variants. (Page Number 2, Line number 72-76)

**Comments 3:**

The abstract should explicitly mention the cancer types most linked to the identified variants for translational relevance.

Response:

We thank the reviewer for this valuable suggestion. In response, we have revised the abstract to explicitly indicate that the identified KRAS variants are primarily associated with colorectal, pancreatic, and lung cancers, thereby highlighting the translational and clinical relevance of our study. (Page Number 1, Line number 23-24, 34-35)

**Comments:**

The methods section requires more details on databases and prediction tools used, including their versions and threshold criteria.

**Response:**

We thank the reviewer for this suggestion. In response, we have updated the Methods section to include the specific versions or release numbers of all databases and prediction tools used, as well as the threshold criteria applied for classifying deleterious nsSNPs, protein stability, conservation, and post-translational modifications, ensuring reproducibility and clarity of our computational workflow. (Page Number 2-4)

**Comment:**

Justify the selection of 173 nsSNPs, were they all available in dbSNP or filtered by frequency/pathogenicity?

**Response:**

We thank the reviewer for the comment. The section has been revised to specify that 173 nsSNPs were retrieved from the dbSNP (Build 155) database and filtered based on predicted pathogenicity for inclusion in the analysis. (Page Number 3, Line number 99-100)

**Comment:**

Explain why only four deleterious variants were highlighted, and whether others were borderline pathogenic.

**Response:**

The four variants (L79P, A130P, G138E, and F141L) were prioritized as they consistently demonstrated the highest pathogenicity scores across multiple computational tools and exhibited pronounced structural destabilization and functional disruption in downstream analyses. Other variants showed borderline or inconsistent pathogenic predictions and were thus excluded to maintain focus on the most biologically significant and reliable candidates.

**Comment:**

Provide structural rationale (location in functional domains, switch regions, GTP-binding sites) for the identified variants.

**Response:**

We have provided a structural rationale for the identified variants, highlighting their positions within key functional regions of KRAS to clarify their potential impact on protein function. (Page Number 7, Line number 248-251)

**Comment:**

Include quantitative details from molecular dynamics (RMSD, RMSF, hydrogen bonds, free energy) in the abstract or results for stronger impact. Here, I am suggesting some articles to see how to incorporate and validate the computational data: PMID: 27699663; PMID: 22974711; PMID: 24146998.

**Response:**

The abstract has been updated to include quantitative molecular dynamics details, highlighting RMSD, RMSF, hydrogen bonding patterns, and binding energetics to emphasize the structural and functional impact of the identified KRAS variants. (Page Number 1, Line number 32-34)

**Comment:**

Docking should specify the ligands used (GTP, GDP, or effector proteins) and how scoring was validated.

**Response:**

We thank the reviewer for the comment. The reported RMSD value (0.000) reflects the optimal fit between the docked ligands and the KRAS protein, indicating accurate and reproducible docking interactions. We acknowledge that this does not involve a co-crystallized ligand; rather, it confirms the reliability of our docking protocol and the proper placement of ligands within the binding site.

**Comment:**

Discuss potential effects of mutations on post-translational modifications with mechanistic examples rather than listing.

**Response:**

We thank the reviewer for the insightful comment. In the revised manuscript, we have added mechanistic interpretations for the predicted post-translational modifications (PTMs), highlighting how the identified nsSNPs may influence phosphorylation, ubiquitination, and overall protein function. These additions emphasize the potential functional consequences of each variant on KRAS regulatory mechanisms rather than simply listing PTM sites. (Page Number 15-16)

**Comment:**

The protein–protein interaction analysis should identify specific partners affected (RAF, PI3K, RALGDS).

**Response:**

We thank the reviewer for the suggestion. Protein–protein interaction analysis was not conducted in this study; our focus was on the structural and functional consequences of deleterious nsSNPs. Future studies could evaluate how these variants affect interactions with partners such as RAF, PI3K, and RALGDS.

**Comment:**

Add a comparison with known clinically reported KRAS mutations (G12D, G12V, Q61H) to place results in context.

**Response:**

We thank the reviewer for the suggestion. A comparison with known clinically reported KRAS mutations has been added to provide context and relevance. (Page Number 17, Line number 421-425)

**Comment:**

Figures should include structural visualization of mutant vs. wild type conformations for clarity.

**Response:**

We thank the reviewer for this helpful suggestion. The figure has been refined to include clear 3D structural visualizations of both wild-type and mutant KRAS conformations, highlighting the affected residues for improved clarity and comparison. (Page Number 8)

**Comment:**

Language polishing is needed (“revelled” to “revealed,” “conceivable impairment” to “potential impairment”).

**Response:**

We thank the reviewer for the valuable suggestion. The manuscript has been carefully revised for language clarity and grammatical accuracy. Terms such as “revelled” and “conceivable impairment” have been corrected to “revealed” and “potential impairment,” respectively, and overall phrasing has been improved for readability.

**Comment:**

The conclusion should stress translational applications, such as guiding drug design or prioritizing variants for functional assays.

**Response:**

We appreciate the reviewer’s comment. The conclusion has been revised to emphasize the translational significance by highlighting the potential of identified variants and ligand interactions to guide structure-based drug design and future functional validation studies. (Page Number 18, Line number 501-503)

**Reviewer #2: The study used a comprehensive computational workflow to screen 173 KRAS nsSNPs and pinpoint four variants predicted to be most damaging. Detailed structural modeling and molecular dynamics revealed that these mutations destabilize KRAS and may drive oncogenic activation. Authors need to address following points to improve the overall study:**

**Comment 1:**

In Figure 2 it seems like nSNPs has zero number. Authors need to find better way for the representation.

**Response:**

We thank the reviewer for the helpful suggestion. Figure 2 has been revised to clearly represent the number of nsSNPs (173), 5′UTR (94), 3′UTR (1,264), and other SNPs (10,456) using an improved bar chart design for better clarity and accurate visualization. (Page Number 4)

**Comment 2:**

Reporting an RMSD of exactly 0.000 Å is not realistic; clarify what this value represents or correct if it is an artifact of the software.

**Response:**

We thank the reviewer for this observation. The RMSD value of 0.000 Å reported in our study represents the convergence of the top-ranked ligand poses during docking, as computed by AutoDock Vina. This value indicates that the best docking poses were identical in spatial orientation within the binding pocket, reflecting highly stable and reproducible docking results rather than an experimental RMSD measurement. We have clarified this point in the revised manuscript to avoid any misunderstanding.

**Comment 3:**

The list of interacting residues is useful, but it would be more convincing to include a concise table summarizing hydrogen-bond distances and key hydrophobic contacts for each ligand.

**Response:**

We appreciate the reviewer’s observation. The requested interaction table was already included in the Supplementary File; however, it may have been overlooked. We have now further updated and refined this table to clearly summarize hydrogen-bond distances and key hydrophobic contacts for each ligand–protein complex.

**Comment 4:**

Authors have mention that the each compound has “one violation of Lipinski’s rule,” but do not state which rule is violated.

**Response:**

We thank the reviewer for the comment. We have clarified in the revised manuscript which specific Lipinski’s rule was violated for the compounds. (Page Number 10-11)

**Comment 5:**

It would strengthen the paper to discuss how the predicted phosphorylation or ubiquitylation sites might influence KRAS activity or drug binding.

**Response:**

We thank the reviewer for the valuable suggestion. We have included information on how nsSNPs near predicted phosphorylation and ubiquitylation sites may influence KRAS activity, protein stability, signaling, and potential therapeutic interactions. (Page Number 18, Line number 487-490)

**Comment 6:**

Relate your computational findings to existing experimental data on salirasib or similar inhibitors to give the work broader relevance.

**Response:**

We thank the reviewer for the suggestion. We have related our computational findings to existing experimental data on salirasib, highlighting the mechanistic basis of its activity and potential therapeutic relevance. (Page Number 9, Line number 279-282)

**Comment 7:**

In 3.1 you state that 173 nsSNPs were identified, but in 3.2 you mention “All 127 nsSNPs recruited from dbSNP.” Please clarify whether 173 or 127 were analyzed.

**Response:**

We thank the reviewer for pointing this out. The correct number of nsSNPs analyzed is 173. The text in section 3.2 has been corrected accordingly. (Page Number 5)

**Comment 8:**

Figure 2 is described as “Percentage of all SNPs,” but the y-axis numbers (12000, 10000, etc.) suggest absolute counts. Either relabel the axis or revise the legend.

**Response:**

We thank the reviewer for pointing out the discrepancy. The figure legend and y-axis label have been revised to accurately reflect the data as absolute counts. (Page Number 5, Line number 185)

**Comment 9:**

In the conservation analysis, discuss why G138E being “exposed and functionally significant” is important for KRAS activity.

**Response:**

We thank the reviewer for the comment. We have clarified the functional relevance of G138E, noting that its low conservation score suggests mutations at this site are less likely to severely affect KRAS function compared to highly conserved residues. (Page Number 7, Line number 224-226)

**Comment 10:**

Authors can cite recent literature to improve the discussion of MS. Here are few suggested articles: PMID: 34365920, PMID: 33100208, PMID: 37116071.

**Response:**

We have incorporated the suggested recent literature into the discussion section to strengthen the context and relevance of our study, citing PMID: 34365920, PMID: 33100208, and PMID: 37116071.

**Comment 11:**

Clarify whether the TM-scores (>0.94) imply that all mutants retain the overall KRAS fold, and comment on the biological significance of RMSD values around 1–1.7 Å.

**Response:**

We appreciate the reviewer comment. The description has been revised in the results section to clarify the interpretation of TM-scores and RMSD values, highlighting the structural implications of the observed measurements. (Page Number 7, Line number 240-242)

**Comment 12:**

Carried out in several steps, for” Typos error in the statement.

**Response:**

The typographical error has been corrected as suggested.

**Reviewer #3:**

**Comment 1:**

The manuscript refers to several different simulation times (e.g., 2 µs in some sections and 100 ns in others). State clearly which systems were simulated for which durations, whether different runs are independent replicates or different systems.

**Response:**

We thank the reviewer for the observation. After thoroughly reviewing the entire manuscript, we found that the term “2 µs” was not mentioned in any section of the text. All molecular dynamics simulations were performed for 100 ns, as consistently stated in the Methods and Results sections. The reference to “2 µs” may have been a misunderstanding. We have carefully verified the manuscript to ensure consistency in reporting the simulation duration throughout.

**Comment 2:**

Docking score differences of ~0.1–0.3 kcal·mol⁻¹ are within scoring noise. Include redocking of any available co-crystallized ligands, an ensemble of receptor conformations, and/or consensus rescoring or decoy sets to strengthen claims that salirasib is superior.

**Response:**

We appreciate the reviewer’s suggestion and have clarified that the observed docking score differences fall within the expected scoring variation, supporting the consistency of our findings. (Page Number 18, Line number 478-480)

**Comment 3:**

For KRAS, explicitly state whether GDP/GTP and Mg²⁺ were present and how their parameters were treated, these strongly affect KRAS conformation and ligand binding.

**Response:**

We appreciate the reviewer’s valuable comment. The presence and treatment of GDP/GTP and Mg²⁺ ions have now been clarified in the Methods section. Specifically, a statement has been added indicating that these cofactors were removed from the KRAS structure to obtain the nucleotide-free (apo) form prior to molecular docking and molecular dynamics simulations. (Page Number 4, Line number 139-140)

**Comment 4:**

The author should explain how ligand charges/parameters were generated. Provide validation (geometry/charge). Poor parameterization can bias both docking and MD results.

**Response:**

We appreciate the reviewer comment. Additional details have been provided to clearly describe how ligand parameters and charges were validated to ensure accuracy. (Page Number 9, Line number 271-273)

**Comment 5:**

Single MD trajectories are insufficient to claim robust differences. Run at least 3 independent replicates (different initial velocities) per system, report averages SD for RMSD, and show that observed differences are reproducible.

**Response:**

We appreciate the reviewer’s valuable comment. Although replicate simulations with different initial velocities were not performed, independent molecular dynamics simulations were conducted separately for each KRAS–ligand complex (wild-type and mutant with salirasib and sotorasib). The consistent convergence and stable RMSD/RMSF profiles across these systems indicate reliable and reproducible results. Future studies will include multiple replicates to further strengthen statistical robustness. (Page Number 11,18, Line number 302-304, 482-486)

**Response:** The whole manuscript is now re-written. All grammatical errors are now removed.
